# Numerical Simulation of Copper-Aluminum Composite Plate Casting and Rolling Process and Composite Mechanism

**DOI:** 10.3390/ma15228139

**Published:** 2022-11-16

**Authors:** Qinghua Chang, Peikai Gao, Junyi Zhang, Yiqang Huo, Zheng Zhang, Jingpei Xie

**Affiliations:** 1 College of Materials Science and Engineering, Henan University of Science and Technology, Luoyang 471023, China; 2 Provincial and Ministerial Co-Construction of Collaborative Innovation Center for Non-Ferrous Metal New Materials and Advanced Processing Technology, Henan University of Science and Technology, Luoyang 471023, China

**Keywords:** copper-aluminum composite plate, liquid-solid casting and rolling, metallurgical bonding, finite element simulation

## Abstract

This paper uses ANSYS Workbench platform to simulate the casting and rolling composite process, taking the horizontal type casting and rolling machine as the research object, and conducts the numerical simulation study of copper-aluminum composite solid-liquid casting and rolling heat-flow coupling, mainly to study different walking speed, aluminum pouring temperature, casting and rolling zone length, heat transfer coefficient on the temperature field, liquid phase rate influence law, and use it as a theoretical guide for copper-aluminum solid-liquid casting. The experiments of copper-aluminum solid-liquid casting-rolling composite were carried out to optimize the process parameters and to verify the experiments, so as to prepare a well-bonded copper-aluminum composite plate. The composite mechanism in the preparation of copper-aluminum composite plate was analyzed, and it was clarified that the interfacial layer was formed through four stages: contact between copper and aluminum surfaces, contact surface activation, mutual diffusion of copper and aluminum atoms, and reaction diffusion.

## 1. Introduction

With the social progress and the continuous development of high-end equipment manufacturing industry, people’s requirements for the mechanical properties of metal materials are gradually improved, and composite materials with various characteristics emerge as the times require. Lightweight structure, functional integration and cost reduction are the new development directions of today’s manufacturing industry. Composite materials made of dissimilar metals according to certain methods are widely used in the design and manufacture of high-end products because of their advantages of giving full play to their respective characteristics and reducing production costs [1,2,3,4]. Copper-aluminum composite made of light weight, low cost, good heat dissipation and high strength, good electrical and thermal conductivity copper has become the focus and hot spot in the material field at present, because it can give full play to the advantages of copper and aluminum. At the same time, the preparation technology, processing technology, application and related composite theory, synergetic deformation theory and other aspects of copper-aluminum laminated composite have become the hot spot of current research [5,6,7,8,9,10]. The results show that compared with pure copper, the weight of Cu-Al composite can be reduced by 40–50% and the cost can be reduced by 30–50% [11], so it has a very wide application prospect in the fields of electric power, heat, information, and machinery [12,13].

The cast-roll method has been widely used for the preparation of single metals such as steel, titanium, aluminum, and magnesium, while for composite materials, it is prepared by involving a second metal on the rolls and using the heat-flow coupling of temperature and force fields to form an interfacial layer between the two metals to achieve metallurgical bonding. For the liquid-solid casting and rolling composite process, in its casting and rolling composite process, the liquid phase metal enters the rolls from the molten pool and undergoes solidification and hot rolling process between the rolls. Casting and rolling area can be divided into three areas: liquid phase area, liquid-solid phase area, solid phase area. The casting and rolling process casting and rolling speed (walking speed), liquid phase pouring temperature, casting and rolling zone length, heat transfer coefficient, and other parameters have an important impact on the temperature field of the casting and rolling zone, liquid phase rate distribution, which directly affects the success of the casting and rolling compound.

This paper takes the horizontal casting and rolling machine as the research object, conducts the numerical simulation study of copper-aluminum composite solid-liquid casting and rolling heat-flow coupling, mainly studies different walking speed, aluminum liquid pouring temperature, casting and rolling zone length, heat transfer coefficient on the temperature field, liquid phase rate influence law, and as a theoretical guide for copper-aluminum solid-liquid casting and rolling composite experiments, optimizes the process parameters, conducts experimental verification, and prepares a good combination of copper-aluminum composite plate. Analysis of the composite mechanism in the preparation of copper-aluminum composite plate. The casting and rolling method of copper-aluminum composite plate has excellent mechanical properties and cost advantages, and has wide application prospects. However, various technological parameters of the preparation process, such as billet speed and pouring temperature, need to be accurately controlled, otherwise it will cause the defects of leakage, uncomposite and low bonding strength. The numerical simulation method has the characteristics of low cost, easy control, and high efficiency. It is necessary to carry out numerical simulation of casting and rolling process of Cu-Al composite plate and study the composite mechanism.

## 2. Solid-Liquid Casting and Rolling Composite Multi-Field Coupling Numerical Simulation

### 2.1. Solid-Liquid Casting and Rolling Composite Process Overview

There are various preparation methods of bimetallic composites, which can be divided into three categories according to the composite state of the base metal components [14], including solid-solid composite method, solid-liquid composite method, and liquid-liquid composite method [15]. Solid-liquid composite technology is widely used in the preparation of copper/aluminum bimetallic composites because of its simple process, little influence by the shape and size of the parts to be composite, and low requirements for equipment. Solid-liquid composite technology includes: composite casting [16], hot dip plating [17], solid-liquid casting-rolling composite [9], etc. Copper/aluminum bimetallic composites have various preparation processes, including rolling [18,19], extrusion [20], drawing [21], cold forging [22], explosive bonding [23], hot dip plating [17], composite casting [16], casting and rolling [24,25], and core filling [26,27].

**Figure 1:**
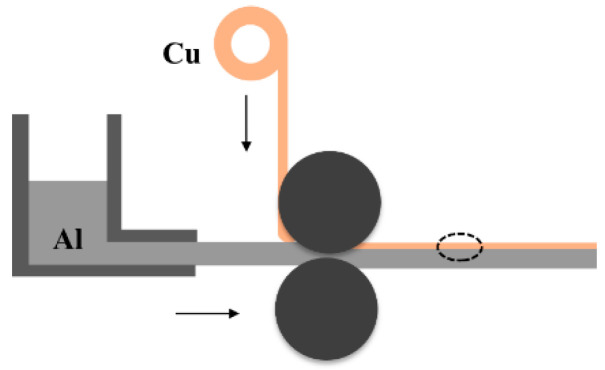
Principle of Cu-Al composite plate casting rolling process.

The use of horizontal two-roller casting and rolling experimental machine for its casting and rolling composite, the process is shown in Figure 1, Industrial pure aluminum ingots are first heated and smelted in the aluminum pond, and then the liquid aluminum enters the filter box to remove the inclinations, and then the clean liquid aluminum enters the front box slowly through the flow trough and stands for a few minutes, so that the slag is deposited, improve the uniformity, and effectively control the pouring temperature, through the flow trough into the casting nozzle, the casting nozzle after the diversion and the surface contact with the pretreated copper strip, into the casting rolling area. Copper strip through the roll to the casting and rolling area and aluminum contact heat exchange, the reaction, in this process, the cooling water in the roll will take away excess heat, aluminum liquid from the liquid phase area by solid-liquid coexistence into solid phase, high temperature aluminum and copper in the heat and mass transfer, the formation of the interface layer to make it achieve metallurgical bonding, by the roll casting and rolling preparation of copper aluminum composite plate.

### 2.2. Basic Assumptions and Basic Control Equations

Copper-aluminum composite plate casting and rolling preparation process is the first aluminum ingot melting made of high-temperature aluminum liquid, aluminum liquid through the sprue into the roll and copper strip under the action of mill pressure, heat and mass transfer, and finally achieve metallurgical bonding. In the preparation process, a large amount of heat is taken away by the cooling water, the basic assumptions for the establishment of the model: aluminum liquid for incompressible fluid, the flow field for the continuity of the steady-state flow field, ignore the copper strip in the rolling process, ignore the deformation of copper and aluminum interface at the solid solution and intermetallic compounds heat transfer, the cooling water circulation in the roll sleeve for the steady-state convective heat transfer, do not consider the copper and aluminum composite plate transverse heat transfer, simplified to a two-dimensional model.

According to the principle of solidification and melting model solution, the energy equation is analyzed step by step. The enthalpy energy of aluminum liquid is obtained from the sum of the enthalpy (*h*) and latent heat content (ΔH).

(1) H=h+ΔH

among

(2) $$h=h_{ref}+\int_{T_{ref}}^{T}c_{p}dT$$

In the formula, $h_{ref}$ for reference enthalpy, $T_{ref}$ for reference temperature, $c_{p}$ for the specific heat under a certain pressure.

Define the liquid phase ratio of the liquid portion β, the value is as follows:

(3) $$\beta =0\;\;\mathrm{if}\;T<T_{solidus}$$

(4) $$\beta =1\;\;\mathrm{if}\;T>T_{liquidus}$$

(5) $$\beta =\frac{T-T_{solidus}}{T_{liquidus}-T_{solidus}}\;\mathrm{if}\;T_{liquidus}>T>T_{solidus}$$

In experience, the latent heat content can be expressed by the solidified latent heat (*L*) of the material:

(6) ΔH=βL

After applying the solidification and melting model, the energy equation is as follows:

(7) $$\frac{\partial }{\partial t}(\rho H)+\nabla •(\rho \vec{v}H)=\nabla •(k\nabla T)+S$$

In formula, *H* is the enthalpy, *ρ* is the density, $\vec{v}$ is the velocity of aluminum liquid, *S* is the generalized source term, the left is the input energy, and the right is the output energy.

### 2.3. Material Parameters

Consistent with the test process, 1050 Al was used for aluminum, T2 Cu for copper, and 32Cr3Mo1V for the roller sleeve, and the physical parameters of each material are shown in Table 1, and the latent heat of solidification was 397.92 J/g.

### 2.4. Geometric Model

**Figure 2:**
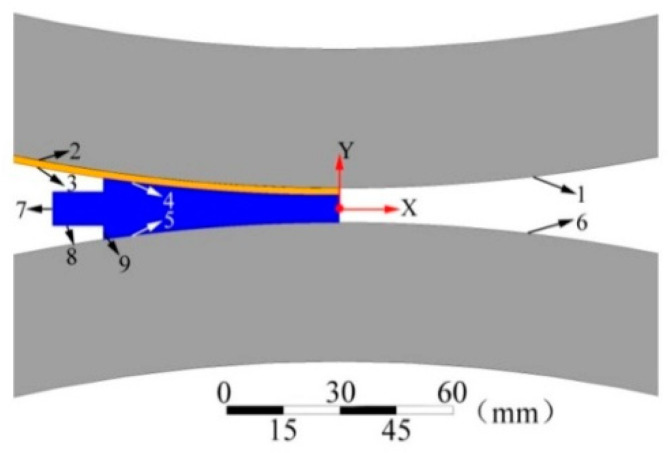
Geometric model of horizontal casting and rolling of copper aluminum composite plate.

The casting and rolling process is modeled using the Design Modeler of the ANSYS Workbench platform, and the schematic diagram of the model is shown in Figure 2.

As can be seen from Figure 2, it mainly includes four regions, which are the upper roll sleeve, copper belt, aluminum liquid, and the lower roll sleeve of the roll. The boundaries are indicated by numbers 1–6, where 1 indicates the part of the upper roll sleeve in contact with the copper belt, 2 indicates the part of the copper belt in contact with the upper roll sleeve, 3 indicates the part of the copper belt in contact with the aluminum liquid, 4 indicates the part of the aluminum liquid in contact with the copper belt, 5 indicates the part of the aluminum liquid in contact with the lower roll sleeve, and 6 indicates the part of the lower roll sleeve in contact with the aluminum liquid. The inner diameter of the roll sleeve is 920 mm, the thickness is 40 mm, simulated casting and rolling of 5 mm thick copper-aluminum composite plate, the thickness of its copper strip is 0.5 mm, and the height of the exit position of the casting and rolling plate is 5 mm in line with the actual production, the main reason for simulating the preparation of 5 mm thick copper-aluminum composite plate is its large market demand, the preparation process is more difficult to control, and the number of rolling is reduced in the process of preparing copper-aluminum composite foil by rolling on copper-aluminum composite plate at a later stage.

### 2.5. Boundary Conditions

The boundary conditions generally refer to the geometric conditions such as the shape and size of the object of study, the physical parameters of the heat transfer object, the initial temperature distribution on the boundary, and its heat transfer with the surrounding medium. In the casting and rolling process, the copper strip and the upper roll sleeve, aluminum and copper strip, aluminum and the lower roll sleeve belong to the heat conduction; for heat conduction, mainly Fourier’s law of heat conduction is followed. Heat transfer between the inner surface of the roll and the flowing cooling water in the roll sleeve belongs to forced convection heat transfer, which mainly follows Newton’s law of cooling, see Equation (8).

(8) $$k_{1}\frac{dT}{dx}=h_{w}\left(T-T_{B}\right)$$

where *k*_1_, roller sleeve thermal conductivity; *h_w_*, forced convection heat transfer coefficient; *T*, roller sleeve wall temperature; *T_B_*, circulating cooling water temperature; for forced convection heat transfer coefficient, its value can be determined by Equation (9).

(9) $$\frac{h_{e}•D}{k_{w}}=0.023{\left(\frac{Dv_{w}}{\eta }\right)}^{0.8}{\left(\frac{c_{w}\eta }{k_{w}}\right)}^{0.4}$$

where *D*, the equivalent diameter of cooling water (m); *k_w_*, the thermal conductivity of cooling water (W·m^−1^·K^−1^); $v_{w}$, the flow rate of cooling water (kg·m^−2^·s^−1^); $c_{w}$, the specific heat capacity of cooling water (J·kg^−1^·K^−1^); *η*, the dynamic viscosity of cooling water (kg·m^−1^·s^−1^).

## 3. Analysis of Simulation Results

Multi-field coupling simulation is a macroscopic physical simulation, which is applicable to the interaction of different physical fields. At present, it is recognized that there are four physical fields in nature, which are electromagnetic field, force field, temperature field, and flow field. There is no single physical field, and various occasions in nature are actually coupling between two or more of the above four physical fields. For example, electromagnetic field-temperature field coupling, flow field-temperature field coupling, temperature field-force field coupling, and other multi-field coupling methods. 

By establishing a model to study the multi-field coupling in the preparation process of copper-aluminum composites, one can provide a theoretical basis and important reference for the actual preparation process. Jin Zhumei et al. [28] used finite elements to establish a model to study the casting and rolling process whose temperature distribution in the temperature field, stress magnitude in each region of the stress field, and fluid flow state, and the study showed that there is a circular flow in the melt pool, which can play a good role in enhancing heat and mass transfer by passing through the stirred flow field region. Huang Huagui et al. [29] used fluent finite element simulation software to establish a coupled heat-flow model of the copper-aluminum casting and rolling preparation process, which was combined with experiments to obtain the distribution law of the KISS point, the liquid-phase rate of the flow field, and the temperature distribution law of the temperature field in the copper-aluminum liquid-solid preparation process, which showed that the location of the KISS point will increase with the preheating temperature of the copper plate and the casting and rolling speed becomes larger. The most suitable location of the KISS point is located at one-half to two-thirds of the height of the melt pool, through the analysis of the copper-aluminum composite plate interface diffusion found that the rolling pressure and aluminum pouring temperature on the copper-aluminum composite plate interface bonding has a certain impact. Ye Lifen et al. [30] made a secondary development of the model when establishing the copper-aluminum liquid solid casting and rolling process, and studied the effects of process parameters such as copper-aluminum casting and rolling speed and aluminum pouring temperature on the temperature distribution at the entrance in the casting and rolling temperature field. Liu Xinhua et al. [31] studied the horizontal-type cast-rolled copper-aluminum composite plate by using ProCAST software through the establishment of a multi-field coupling model, and the temperature field of the steady-state process was studied and analyzed to derive reasonable process parameters for the casting and rolling process, and the simulation was verified for its reasonableness by preparing copper-aluminum composite plates using casting and rolling experiments.

### 3.1. Effect of Travel Speed on the Casting and Rolling Process of Copper-Aluminum Composite Plate

#### 3.1.1. The Effect of Different Walking Speed on the Temperature Field Distribution

**Figure 3:**
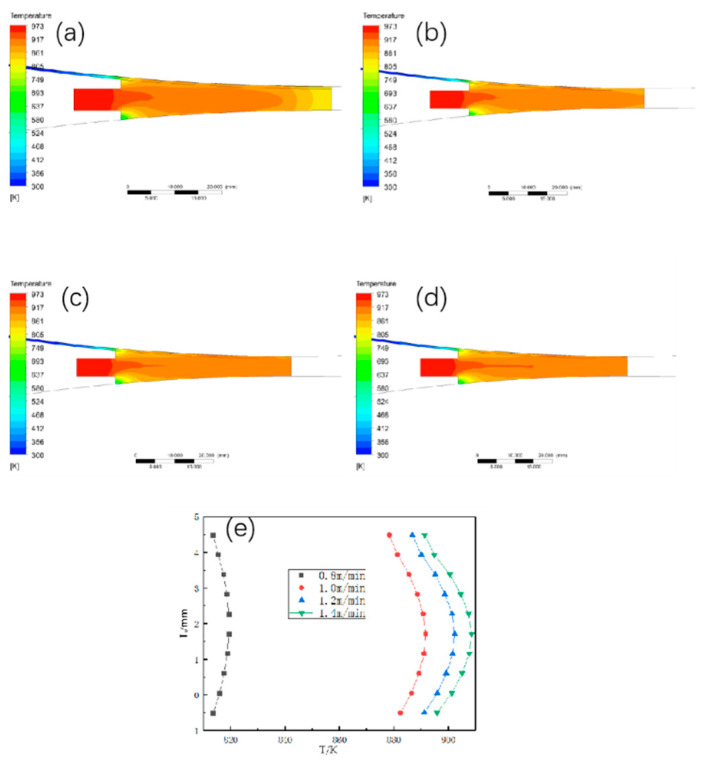
Temperature field and temperature distribution in thickness direction at outlet under different blank walking speeds. (**a**) 0.8 m/min; (**b**) 1 m/min; (**c**) 1.2 m/min; (**d**) 1.4 m/min; (**e**) temperature change curve in thickness direction at outlet.

The temperature field and the temperature at the exit position are shown in Figure 3 when the casting temperature is set to 700 °C, the length of the casting and rolling area is 45 mm, and the heat exchange coefficient is 10,000 w/°C∙m^2^, and the walking speed is changed to 0.8 m/min, 1 m/min, 1.2 m/min, and 1.4 m/min, respectively. The temperature field and the temperature at the exit position increase significantly as the billet speed gradually changes from 0.8 m/min to 1.4 m/min, and when the billet speed changes from 0.8 m/min to 1 m/min, the average temperature at the exit position in the thickness direction increases by 70 °C. When the billet speed changes from 1 m/min to 1.2 m/min and from 1.2 m/min to 1.4 m/min, the temperature at the exit position in the thickness direction only increases by 20 °C. The temperature at the exit position in the thickness direction only increased by 20 °C, and its temperature did not change drastically with the walking speed in the first period, so the change of the walking speed from 0.8 m/min to 1 m/min should not be too fast.

#### 3.1.2. The Effect of Different Billet Speed on the Liquid Phase Rate Distribution

**Figure 4:**
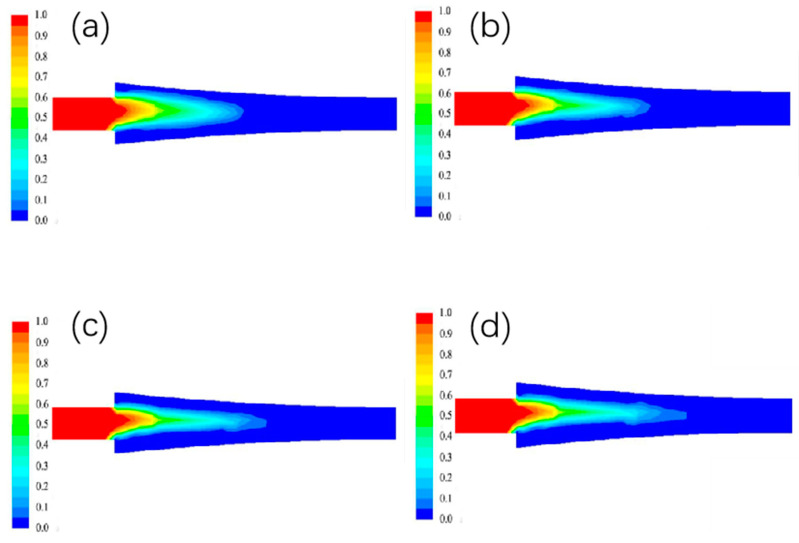
Distribution of liquid phase rate under different blank walking speeds. (**a**) 0.8 m/min; (**b**) 1 m/min; (**c**) 1.2 m/min; (**d**) 1.4 m/min.

The liquid phase rate at different billet speeds is shown in Figure 4. In the figure, blue represents the solid phase, red represents the liquid phase, and other colors represent the solid-liquid zone.The liquid phase rate at the entrance position is lower due to the higher temperature of the aluminum liquid near the copper side, resulting in a thicker billet shell. As the billet walking speed increases, the contact time between the copper strip and the casting and rolling area gradually decreases, so that its heat exchange at the interface gradually decreases, and the width of the liquid phase area becomes narrower and the length increases.

### 3.2. Influence of Casting Temperature on the Casting and Rolling Process of Copper-Aluminum Composite Plate

#### 3.2.1. The Effect of Different Casting Temperatures on the Temperature Field Distribution

**Figure 5:**
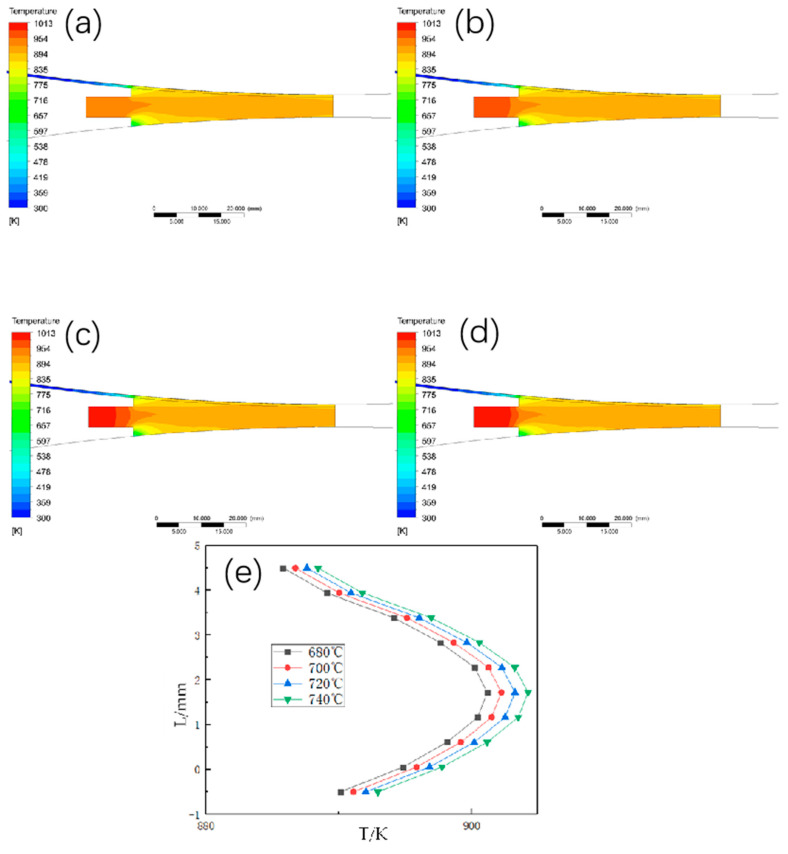
Temperature field at different pouring temperatures and temperature distribution in thickness direction at outlet. (**a**) 680 °C; (**b**) 700 °C; (**c**) 720 °C; (**d**) 740 °C; (**e**) temperature change curve in thickness direction at outlet.

Set the casting speed to 1.2 m/min, the length of the casting and rolling zone to 45 mm, the heat exchange coefficient to 10,000 W/°C∙m^2^. Set the casting temperature to 680 °C, 700 °C, 720 °C, and 740 °C to simulate the temperature field, the simulation results are shown in Figure 5, with the increase of the casting temperature of aluminum liquid, compared with the casting and rolling speed, its influence on the temperature field is smaller.

#### 3.2.2. The Effect of Different Pouring Temperature on the Liquid Phase Rate Distribution

**Figure 6:**
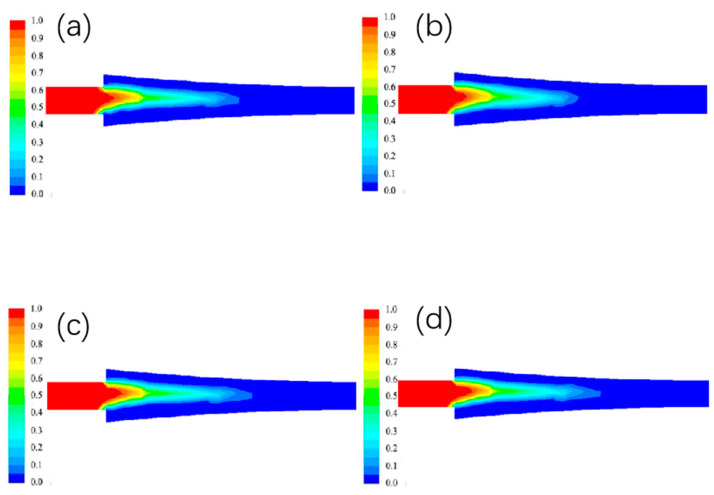
Distribution of liquid phase rate at different pouring temperatures (**a**) 680 °C; (**b**) 700 °C; (**c**) 720 °C; (**d**) 740 °C.

The distribution of liquid phase rate at different pouring temperatures is shown in Figure 6, and the color representation is the same as above. With the increase of pouring temperature, the proportion of liquid phase depth to the length of casting and rolling area is increasing, and the ratio is less than 2/3. However, when the pouring temperature is 720 °C and 740 °C, the liquid phase depth increases, and at this time, the diffusion reaction is more likely to occur at the copper-aluminum interface, and it is easier to achieve metallurgical bonding and reduce the rolling force.

### 3.3. The Effect of the Length of the Casting and Rolling Zone on the Casting and Rolling Process of Copper and Aluminum Composite Plate

#### 3.3.1. The Effect of Different Casting and Rolling Zone Length on the Temperature Field Distribution

**Figure 7:**
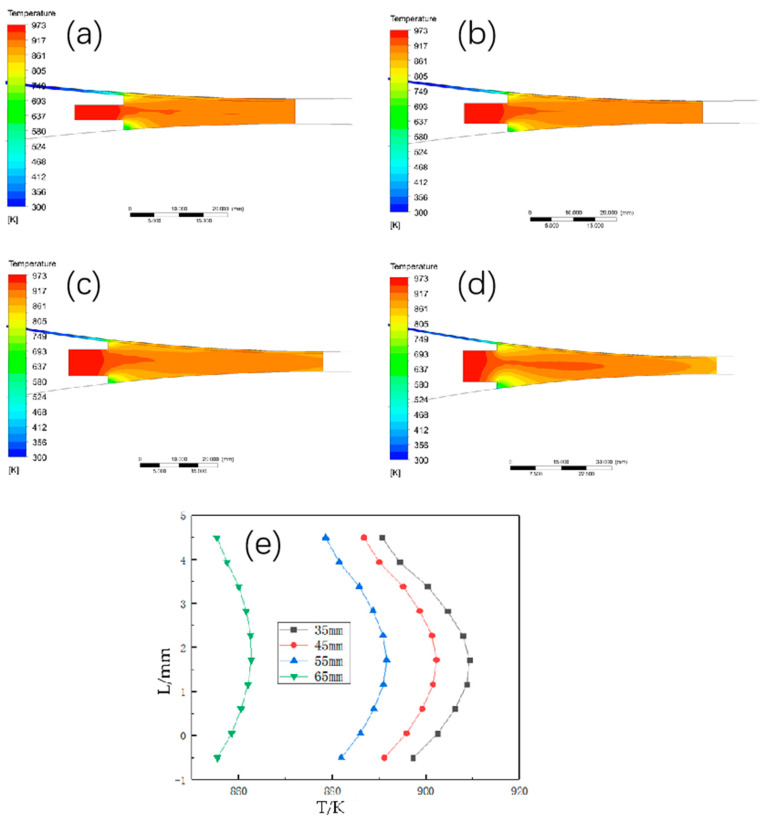
Temperature field and temperature distribution in thickness direction at outlet under different casting and rolling zone lengths. (**a**) 35 mm; (**b**) 45 mm; (**c**) 55 mm; (**d**) 65 mm; (**e**) temperature change curve in thickness direction at outlet.

Set the casting and rolling walking speed of 1.2 m/min, casting temperature of 700 °C, heat exchange coefficient of 10,000 W/°C∙m^2^. Change the length of the casting and rolling zone, respectively 35 mm, 45 mm, 55 mm, and 65 mm, the temperature field distribution is shown in Figure 7, with the increase of the length of the casting and rolling zone, the temperature maximum in the temperature field gradually shifts to the exit and spreads to both sides. As the length of the casting and rolling zone grows, the residence time of the aluminum liquid in the casting and rolling zone becomes longer, the heat exchange intensifies, the heat taken away by the circulating cooling water gradually increases, and the solid phase metal solidifies faster. Exit temperature decreases significantly, the width of the liquid phase area changes significantly, so the length of the casting and rolling area should be increased at the same time to increase the casting temperature or slowly increase the casting and rolling billet speed.

#### 3.3.2. The Effect of Different Casting and Rolling Zone Length on the Liquid Phase Rate Distribution

**Figure 8:**
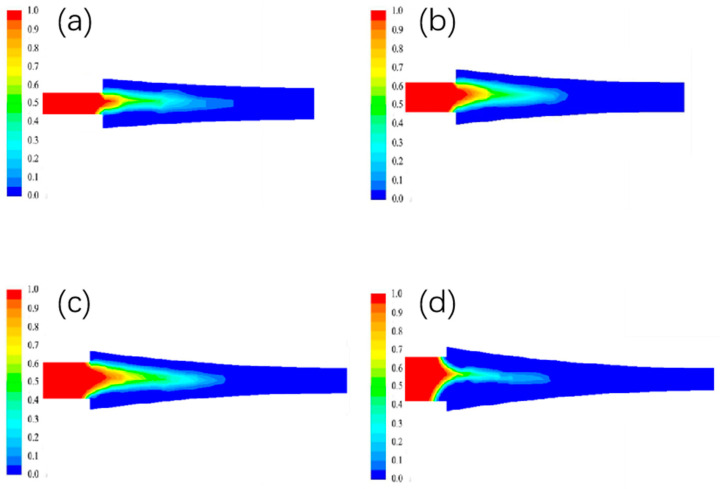
Distribution of liquid phase rate under different casting and rolling zone lengths. (**a**) 35 mm; (**b**) 45 mm; (**c**) 35 mm; (**d**) 45 mm.

The liquid phase rate distribution of different casting and rolling zone lengths is shown in Figure 8, and it can be seen from the figure that the ratio of the liquid phase depth to the length of the casting and rolling zone gradually decreases as the length of the casting and rolling zone increases.

### 3.4. Effect of Heat Transfer Coefficient on the Casting and Rolling Process of Copper-Aluminum Composite Plate

#### 3.4.1. The Effect of Different Heat Exchange Coefficients on the Temperature Field Distribution

**Figure 9:**
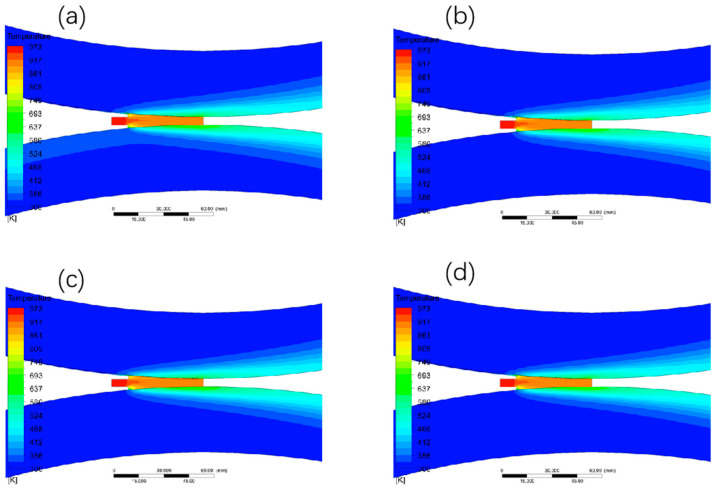
Temperature field and temperature distribution in thickness direction at outlet under different heat exchange coefficients. (**a**) 6000 W/(m^2^·K); (**b**) 9000 W/(m^2^·K); (**c**) 12,000 W/(m^2^·K); (**d**) 15,000 W/(m^2^·K).

Set the walking speed of 1.2 m/min, casting temperature of 700 °C, casting and rolling area length of 45 mm, heat exchange coefficient of 6000 W/(m^2^·K), 9000 W/(m^2^·K), 12,000 W/(m^2^·K), and 15,000 W/(m^2^·K). The temperature field distribution is shown in Figure 9. From the figure it can be seen that the temperature gradually increases with the increase in the convective heat transfer coefficient. The lower the convection heat transfer coefficient, the more obvious the cooling effect is, mainly because the convection heat transfer coefficient directly determines the heat flow density and determines the heat exchange efficiency, the larger the convection heat transfer coefficient, the higher the heat flow density, the higher the heat exchange efficiency and the lower the outlet temperature.

#### 3.4.2. The Effect of Different Heat Transfer Coefficients on the Liquid Phase Rate Distribution

**Figure 10:**
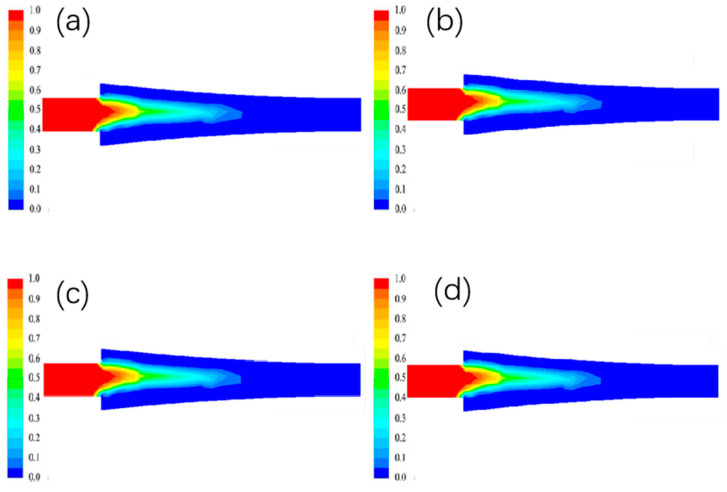
Liquid phase rate distribution under different heat exchange coefficients. (**a**) 6000 W/(m^2^·K); (**b**) 9000 W/(m^2^·K); (**c**) 12,000 W/(m^2^·K); (**d**) 15,000 W/(m^2^·K).

The liquid phase ratio under different heat transfer coefficients is shown in Figure 10. When other conditions remain unchanged, with the change in heat transfer coefficient, as the thickness of the front section of the casting and rolling area is greater than the thickness of the tail section of the casting and rolling area, the cooling of the front end of the casting and rolling area is slower, and for the existence of liquid phase, liquid phase into solid phase to consider the latent heat of solidification, it needs to absorb excess heat to provide latent heat of solidification, in the casting and rolling of copper-aluminum composite plate, as far as possible by adjusting the casting and rolling away from the billet speed, aluminum pouring temperature to control. The appropriate liquid phase depth in the length of the casting and rolling area of the proportion.

## 4. Solid-Liquid Casting and Rolling Experiments

### 4.1. Casting Temperature

**Figure 11:**
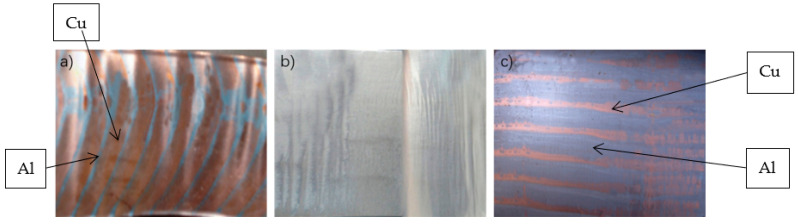
Casting and rolling of copper and aluminum at different casting temperatures with casting speed of 1.2 m/min. (**a**) 670 °C; (**b**) 700 °C; (**c**) 730 °C.

Due to the different expansion coefficients of copper and aluminum, when the casting temperature is too high, there will be internal stress between copper and aluminum, and when the casting temperature is too low, due to the high temperature aluminum liquid to provide energy, it will cause insufficient energy in the process of copper-aluminum casting and rolling compound, so that the compound rate decreases, and the bonding strength of copper-aluminum composite plate decreases. In addition, the casting temperature of aluminum liquid will also affect the size of aluminum strip grain. Figure 11 shows a 5 mm thick, 800 mm wide copper-aluminum composite plate obtained under different temperature conditions, where the thickness of the copper layer is 0.5 mm.

When the pouring temperature is 670 °C, the composite rate of copper and aluminum composite layer is low, about 10–20%, the composite strength is also very low, which is mainly due to the low temperature of aluminum liquid bring in enough energy to make its casting and rolling process to produce metallurgical bonding; when the temperature rises to 700 °C, the composite rate can reach 100%, the bonding strength can reach 15 N/mm. When other conditions remain unchanged, increasing the aluminum liquid pouring temperature will provide more energy to the casting and rolling process to provide enough energy to activate copper and aluminum atoms in the reaction to achieve metallurgical bonding, and also to improve the bonding strength of the composite plate. However, the higher the aluminum pouring temperature, which should not be the case, when the aluminum pouring temperature reaches 730 °C, the aluminum will cause overheating, and the surface will have many pores, which is because, in the aluminum solidification process a lot of air results in defects, hence resulting in the rolling composite failure.

### 4.2. Walking Billet Speed

**Figure 12:**
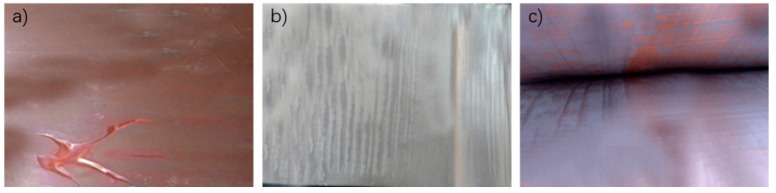
Casting and rolling of copper and aluminum at different casting speeds at pouring temperature of 700 °C. (**a**) 1.1 m/min; (**b**) 1.2 m/min; (**c**) 1.3 m/min.

In casting and rolling, when the billet speed is fast and the aluminum liquid and copper contact time is short, not by enough time for rolling, copper and aluminum metallurgical combination fails; when the billet speed is too slow, and copper and aluminum contact time is too long, it results in semi-molten state control difficulty, high temperature aluminum liquid and copper strip contact for too long, and over-burning and other problems, hence making it difficult for the copper and aluminum to composite. At a aluminum liquid casting temperature of 700 °C, the different walking billet speeds of copper and aluminum composite layer are shown in Figure 12.

As can be seen from Figure 12, when the billet speed is 1.1 m/min, due to the slow speed, the copper strip is too long in the casting and rolling area, and the excessive heat input makes the copper strip overburned; because the casting speed is too slow, the contact time between copper strip and aluminum liquid is long, the aluminum liquid provides more heat, the surface temperature of copper strip is too high, the aluminum liquid solidifies and produces shrinkage holes, which makes the copper and aluminum surface produce a bulge. When the billet speed is 1.3 m/min, due to the copper and aluminum in the casting and rolling area rolling force action time is too short, there is a local uncompounded situation; because the billet speed is too fast, the contact time between copper strip and liquid aluminum is too short, the liquid aluminum does not provide enough energy, and the atoms have no time to diffuse, which reduces the recombination rate. When the billet speed is 1.2 m/min, the compounding situation is good, and the compounding rate can reach 100%, smooth surface, no burr. The suitable rolling speed should be controlled at about 1.2 m/min.

### 4.3. Compounding Mechanism Analysis

The structure and thickness of the interfacial layer of intermetallic compounds produced by the interdiffusion of copper and aluminum are different when the casting temperature, billet speed, and rolling pressure are different, and the reaction of copper and aluminum interdiffusion to form intermetallic compounds consists of four stages [32].

In the first stage, liquid aluminum and solid copper in the roll pressure field and temperature field together, the local physical contact, the slower the walking speed, the longer the time, the larger the contact area.

In the second stage, the temperature of copper surface increases due to contact with high temperature aluminum liquid, and some atoms are activated. In this process, various point defects and line defects such as vacancies and dislocations in the base copper will be generated in this process, and as the contact time between copper and high temperature aluminum liquid increases, the temperature of copper gradually increases, and various crystal defects will become denser with the temperature increase, forming more activation centers, and promoting the reaction to continue.

In the third stage, the activated copper atoms diffuse into aluminum, and the activated aluminum atoms diffuse into copper, forming a mutual diffusion. The aluminum atoms enter into copper after activation and form a solid solution with copper as the solvent and aluminum as the solute; the copper atoms enter into aluminum after activation and form a solid solution with aluminum as the solvent and copper as the solute. At this point, no change in the organization of the interfacial zone can be observed, and due to the low concentration, no new phase is formed yet. The literature shows [33] that after preheating the copper strip, when casting and rolling, as the aluminum liquid encounters the copper strip with a lower temperature will solidify releasing latent heat of solidification, due to the good thermal conductivity of copper, copper will absorb this part of the heat after contact with the high temperature aluminum liquid, and this energy will intensify the vibration of copper atoms, leaving the equilibrium position to form cavities or even destroy the metal bonds between copper atoms to form cracks, reducing the force between copper atoms and activation centers are formed. According to the state of the substrate aluminum in the preparation process, the casting and rolling zone can be divided into semi-solid zone and solid zone; the semi-solid zone is in the front of the casting and rolling zone, and the solid zone is in the back of the casting and rolling zone. In the semi-solid zone, the aluminum liquid is partially solidified for the casting zone, and in the solid zone, the aluminum is basically completely solidified for the rolling zone. For aluminum liquid, due to the difference in density, the volume of the same mass of aluminum liquid is larger than its volume in the solid state when the rolling zone is in the solid state, and the Al–Al bond energy is lower than the Cu–Cu bond energy [34,35], so some of the Al–Al bonding bonds in the aluminum liquid are broken to a greater extent than the Cu–Cu bonds in the copper matrix, which can promote the activation of copper atoms into the aluminum liquid to form solid solution. In addition, under the action of mill pressure, the contact between copper and aluminum atoms generates weak van der Waals forces, and this weak van der Waals force when the pressure is large enough its size is even close to chemical bonding, and the interdiffusion between copper and aluminum atoms forms point bonding, line bonding, and surface bonding under the combined effect of the pressure field formed by the mill rolling and the temperature field provided by the aluminum liquid with heat, and finally solid solution is formed [36].

The fourth stage is reaction diffusion. Copper and aluminum form solid solution in the concentration of a certain size after the formation of intermetallic compounds, copper and aluminum to achieve metallurgical bonding. Later in the casting and rolling process, with the heat dissipation, copper and aluminum composite plate temperature gradually reduced, at this time, the intracrystalline diffusion speed gradually reduced, so that it is significantly lower than the grain boundary diffusion. Due to the existence of interfacial energy, so that when copper atoms and aluminum atoms interdiffuse, the concentration at the interface changes abruptly and reaches the limit solubility of copper and aluminum intermetallic compounds, eutectic reactions will occur, and the newly generated intermetallic compounds will occupy the defects in the formation of solid solution in the early stage, and the phases are redistributed to finally reach equilibrium [37]. Guo Yajie et al. [38] of Chang’an University used the activation sintering method to prepare copper-aluminum laminate composites, and analyzed the interdiffusion of copper and aluminum in the temperature range of 400–500 °C until the intermetallic compounds generated copper and aluminum that reached metallurgical bonding, which showed that the process mainly includes four stages: physical contact of copper and aluminum, nucleation of intermetallic compounds, intermetallic compounds along the interface perpendicular to the interface direction. The first connected the intermetallic compounds along the interface direction to grow the interface thickening. In the copper-aluminum liquid casting and rolling process, the high-temperature aluminum liquid undergoes two processes: partial solidification in the front section and semi-solid hot rolling process in the back section. Because the temperature of the roll sleeve is lower than the temperature of the copper strip preheated by 300 °C, in the process of solidification of liquid aluminum, it is first the aluminum liquid next to the roll sleeve that solidified to form a condensate shell, with the rotation of the mill roll, convective heat transfer continues, the thickness of the condensate shell increasing. In the direction of casting and rolling perpendicular to the copper side gradually solidified.

## 5. Conclusions

This paper analyzes the copper strip pretreatment process, constructs a copper-aluminum casting and rolling model, analyzes the copper-aluminum casting and rolling mechanism, and draws the following conclusions.

(1) When the thickness of the aluminum substrate is 4.5 mm and the thickness of the copper substrate is 0.5 mm after casting and rolling, the better process parameters are: walking speed 1.2 m/min, casting temperature 700 °C, casting and rolling zone length 45 mm, and heat exchange coefficient 10,000 W/(m^2^·K).
(2) The copper-aluminum composite plate with good metallurgical bonding was obtained by treating the copper surface with mechanical polishing method, preheating the copper strip at 300 °C, using 700 °C casting temperature and 1.2 m/min casting speed.
(3) Copper and aluminum metallurgical bonding, the formation of intermetallic compounds need to go through four stages, respectively: contact between copper and aluminum surfaces, contact surface activation, copper and aluminum atoms diffuse each other, reaction diffusion.

## Figures and Tables

**Table 1:** Thermophysical parameters of materials.

T/(K)		300	673	873	923	930	1073
	*c*/(J·kg^−1^·K^−1^)	906	1075	1429	42,100	1172	1173
1050Al	*λ*/(W·m^−1^·K^−1^)	225	218	205	158	90	94
	*μ*/(kg·m^−1^·s^−1^)	100	100	8.323	1.002	0.00133	0.000997
		*ρ*/(kg·m^−3^)		*c*/(J·kg^−1^·K^−1^)		*λ*/(W·m^−1^·K^−1^)	
Roll sleeve		7830		560		31	
Copper T2		8920		386		398	

## Data Availability

Not applicable.
